# Supplementary figures and images for: UV irradiation alters TFAM binding specificity and compaction of DNA
Source: eLife. 2026 Mar 25;14:RP108862. doi: 10.7554/eLife.108862 (PMC13016609; doi:10.7554/eLife.108862)

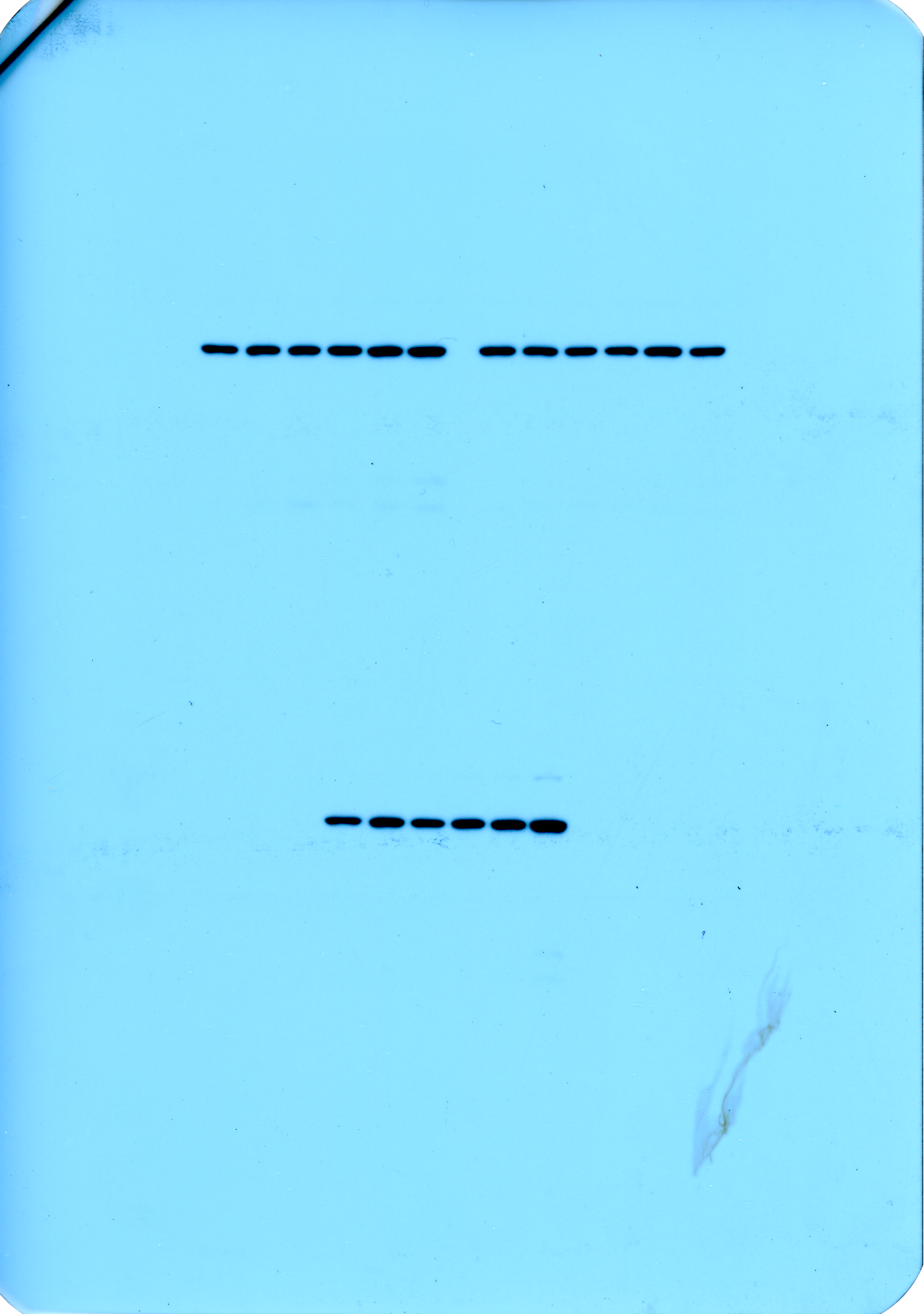

Supplement: Figure 5—source data 1. [file elife-108862-fig5-data1.zip › Figure 5B source data 1/12-18-22 WB TFAM_Bactin reblot 2 001.tif]

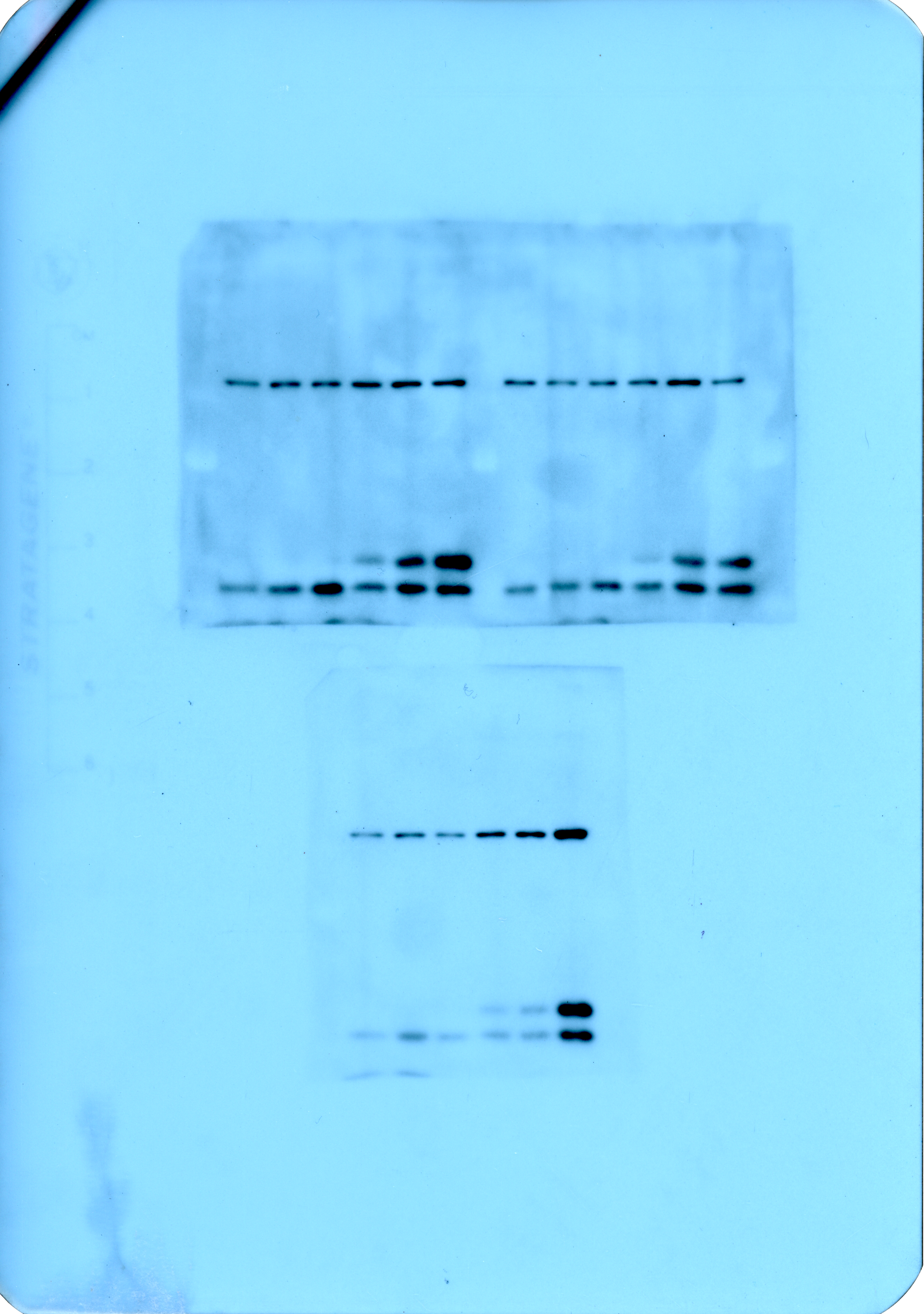

Supplement: Figure 5—source data 1. [file elife-108862-fig5-data1.zip › Figure 5B source data 1/12-16-22 WB TFAM_HeLa extracts 003.tif]

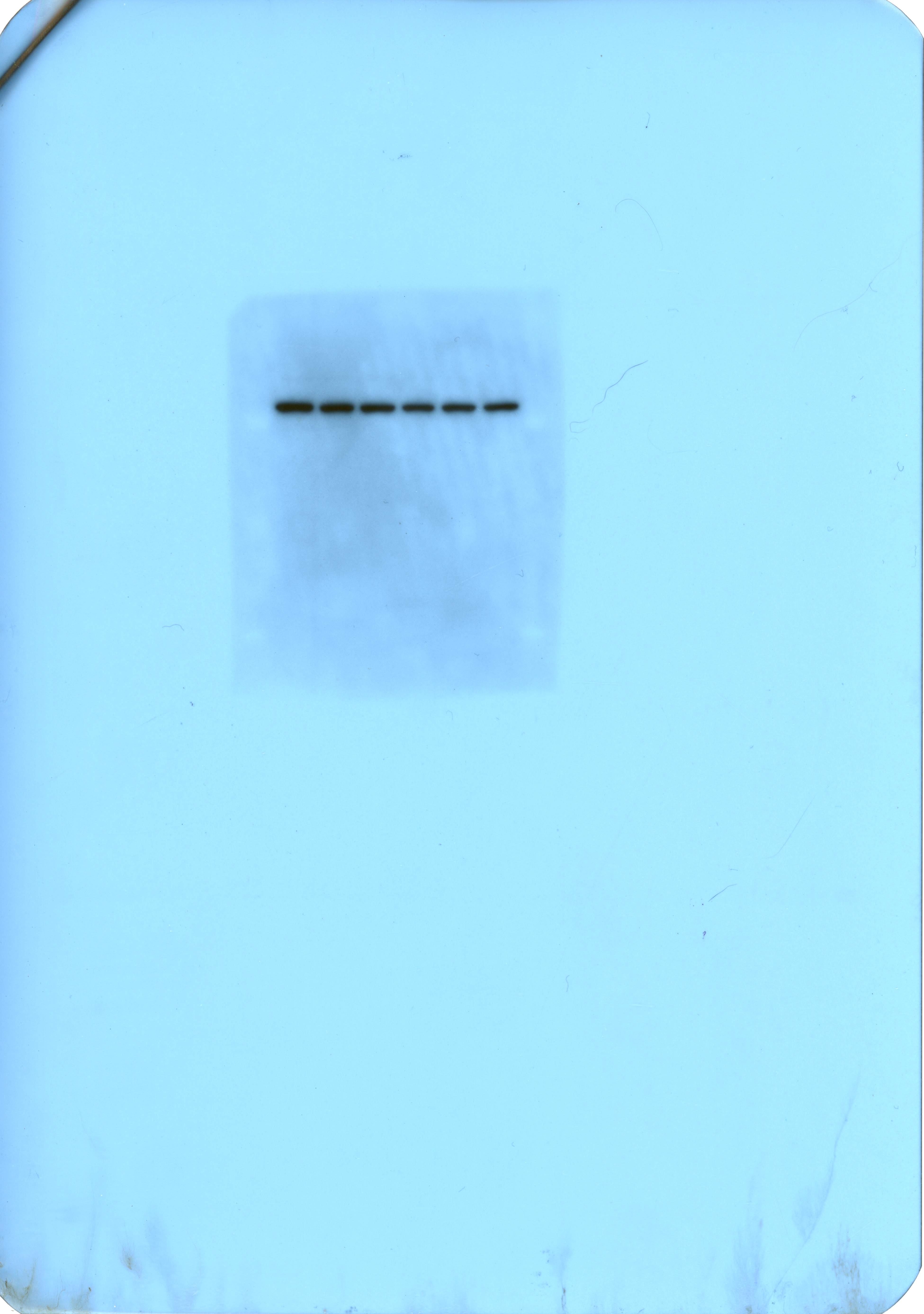

Supplement: Figure 5—source data 1. [file elife-108862-fig5-data1.zip › Figure 5B source data 1/01-02-23 WB TFAM Bactin 001.tif]

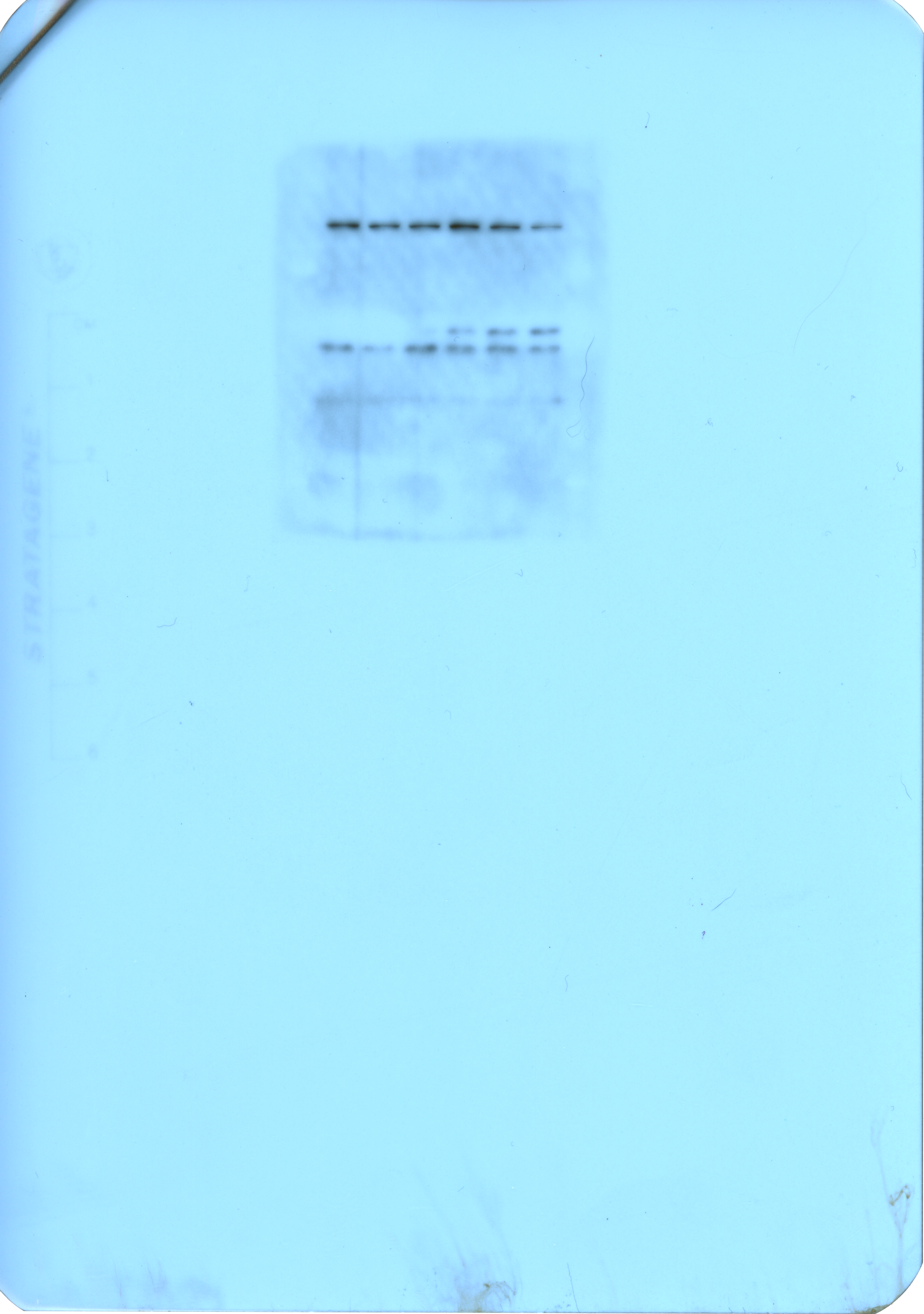

Supplement: Figure 5—source data 1. [file elife-108862-fig5-data1.zip › Figure 5B source data 1/01-01-23 WB TFAM 005.tif]
