# Supplementary material for: UV irradiation alters TFAM binding specificity and compaction of DNA
Source: eLife. 2026 Mar 25;14:RP108862. doi: 10.7554/eLife.108862 (PMC13016609; doi:10.7554/eLife.108862)
Supplement: Figure 5—source data 2. [file elife-108862-fig5-data2.zip › Figure5_Source_Data_2.pdf]

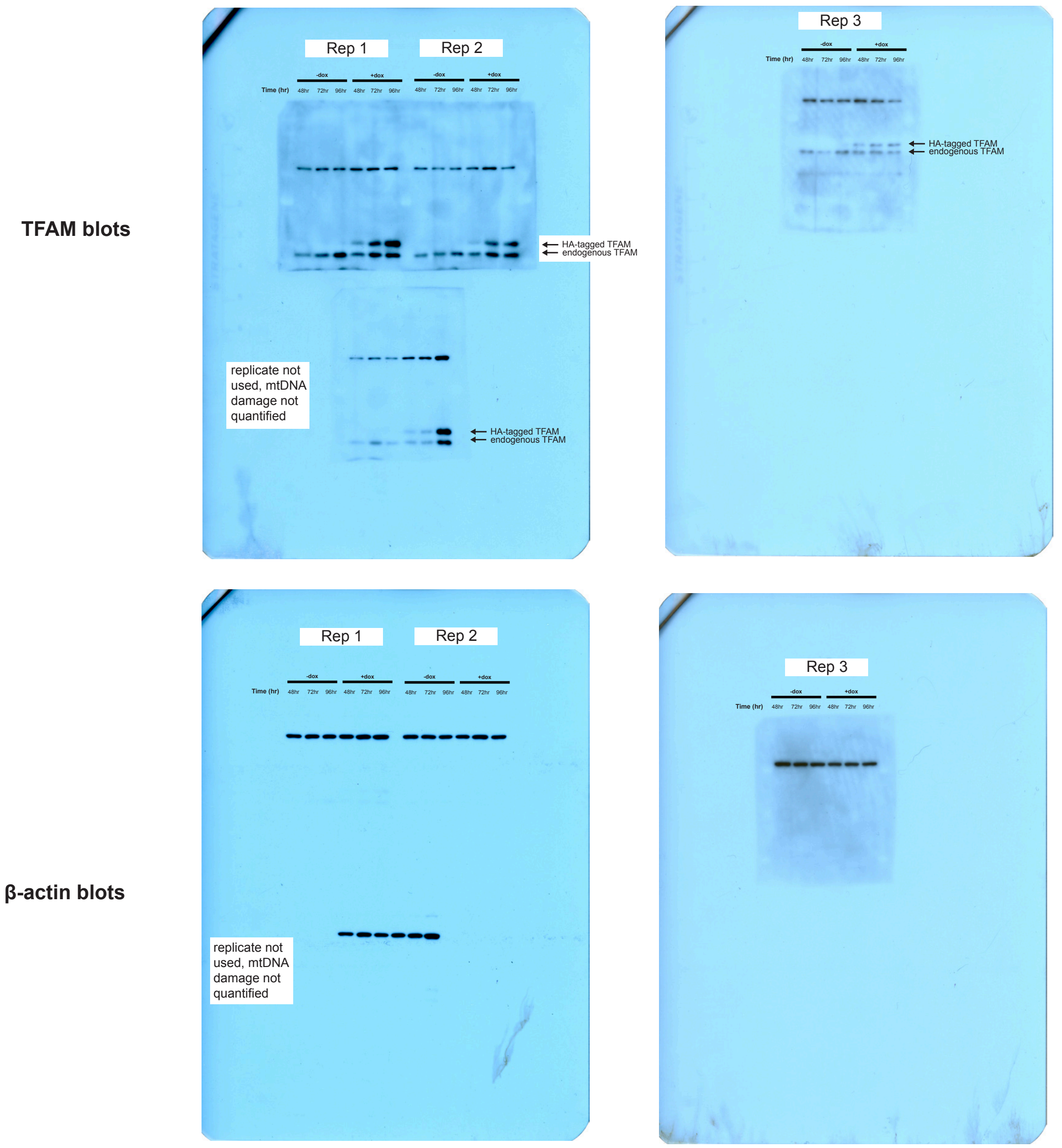

Figure 5, Source Data 2: Original membranes corresponding to Figure 5, panel B. All replicates are shown. The representative image shown in Figure 5B comes from replicate 1. The top membranes correspond to TFAM and the bottom membranes correspond to β-actin. All samples are label with their doxycyline treatment status as well as their time exposed to doxycyline, as outlined in Figure 5A.
