# Supplementary material for: UV irradiation alters TFAM binding specificity and compaction of DNA
Source: eLife. 2026 Mar 25;14:RP108862. doi: 10.7554/eLife.108862 (PMC13016609; doi:10.7554/eLife.108862)
Supplement: Supplementary file 1. [file elife-108862-supp1.docx]

| **Category** | **15nM TFAM -UV** | **15nM TFAM +UV** | **30nM TFAM**  **-UV** | **30nM TFAM**  **+UV** |
| --- | --- | --- | --- | --- |
| Free DNA | 74 | 14 | 4 | 5 |
| Dispersed | 21 | 9 | 29 | 2 |
| Intermediate (tracts) | 36 | 20 | 47 | 19 |
| Punctate | 5 | 23 | 11 | 39 |
| **Total Number of DNAs** | N = 136 | N = 66 | N = 91 | N = 65 |
