# Supplementary material for: UV irradiation alters TFAM binding specificity and compaction of DNA
Source: eLife. 2026 Mar 25;14:RP108862. doi: 10.7554/eLife.108862 (PMC13016609; doi:10.7554/eLife.108862)
Supplement: Supplementary file 2. [file elife-108862-supp2.docx]

| **Oligonucleotide name** | **Sequence (5’ – 3’)** |
| --- | --- |
| ND1_288 | CTC CAC ACT AGC AGA GAC CAA CCG AAC CCC CTT |
| COX2_229 | GCC CCC ATT CGT ATA ATA ATT ACA TCA CAA GAC |
| TRNT_10 | CTT GTA GTA TAA ACT AAT ACA CCA GTC TTG TAA |
| ND2_401 | CAA ATG GGC CAT TAT CGA AGA ATT CAC AAA AAA |
| ND3_92 | TTA GTA GCT ATT ACC TTC TTA TTA TTT GAT CTA |
| ND1_450 | ACT CAC CCT AGC ATT ACT TAT ATG ATA TGT CTC |
| ND6_87 | TTC CTA CAC TAT TAA AGT TTA CCA CAA CCA CCA |
| RNR2_619 | ATT GAT CCA ATA ACT TGA CCA ACG GAA CAA GTT |
| COX1_27 | AAG ACA TTG GAA CAC TAT ACC TAT TAT TCG GCG |
| ND4_473 | CGG CGC AGT CAT TCT CAT AAT CGC CCA CGG GCT |
| ND1_353 40mer | CAC AAA CAT TAT TAT AAT AAA CAC CCT CAC CAC TAC AAT C |
| ND1_353 | ATT ATT ATA ATA AAC ACC CTC ACC ACT ACA ATC |
